# Do Only White or Asian Males Belong in Genius Organizations? How Academic Organizations’ Fixed Theories of Excellence Help or Hinder Different Student Groups’ Sense of Belonging

**DOI:** 10.3389/fpsyg.2021.631142

**Published:** 2021-02-12

**Authors:** Christina Bauer, Bettina Hannover

**Affiliations:** ^1^Department of Psychology, Technische Universität Dresden, Dresden, Germany; ^2^Department of Educational Science and Psychology, Freie Universität Berlin, Berlin, Germany

**Keywords:** stereotypes, organizational implicit theories, gender, ethnicity, belonging, prototype

## Abstract

High-profile organizations often emphasize fixed giftedness rather than malleable effort-based criteria as critical for excellent achievements. With giftedness being primarily associated with White or Asian males, such organizational implicit theories of excellence may shape individuals’ sense of belonging depending on the extent to which they match the *gifted White/Asian male prototype*, i.e., the prototypical gifted person which is typically imagined to be a White or Asian male. Previous research has reported fixed excellence theories emphasizing giftedness (vs. malleable theories emphasizing effort) to impair the sense of belonging of females and negatively stereotyped ethnic minorities. We investigate the combined effects of gender and ethnicity. We predicted that, while individuals whose gender and ethnicity do not match the gifted prototype show a reduced sense of belonging in fixed organizations, White/Asian males who match the gifted prototype show the opposite effect, experiencing a *higher* sense of belonging in fixed (vs. malleable) organizations. In an experimental study (*N* = 663 students), we manipulated advertising material used by a highly selective academic institution in Germany and tested effects on students’ belonging. Whereas the original material emphasized giftedness as essential for excelling (fixed excellence version), our manipulated version stressed effort (malleable version). As expected, females from stereotyped ethnic minority groups felt less belonging in the fixed (vs. malleable) organization, while White/Asian males anticipated stronger belonging in the fixed (vs. malleable) organization. Fixed views of excellence impair negatively stereotyped individuals’ belonging but may even strengthen the belonging of prototypical academic elites.

## Introduction

“*The [organization name] supports young people with a high scientific or artistic giftedness who, [*…*] successfully study [*…*] and from whom, according to their giftedness and personality, special achievements [*…*] are to be expected.*”

Selective organizations like the Ivy League, scholarship providers, or high profile companies are looking for individuals with excellent achievements. The above quote from the advertising material of a highly selective German scholarship provider (which we manipulated in the present study) illustrates how these organizations often inadvertently send messages about their implicit theories of excellence. The quote specifically exemplifies the common *fixed* view of excellence – i.e., the idea that excellent achievements are based on innate and stable personal characteristics, like giftedness or talent, rather than having to be developed through effort (malleable view of excellence). Implicit theories of excellence may, however, not only signal *how* one can reach excellent achievements within an organization, but also *which groups of people* may be likely to do so, thus differentially fostering individuals’ sense of belonging.

Intellectual stereotypes associate giftedness with males rather than females and with White or Asian individuals rather than other non-Asian ethnic minorities (e.g., African or Middle Eastern individuals) who are negatively stereotyped (Fiske et al., 2002; Cuddy et al., 2009; Bian et al., 2017). Based on these stereotypes, fixed organizational excellence theories may not make all individuals belong equally. Indeed, previous research (Leslie et al., 2015; Bian et al., 2018) has found evidence that fixed organizational theories with their emphasis on giftedness may impair the sense of belonging of females and negatively stereotyped ethnic minorities who do not match the gifted White/Asian prototype, i.e., the prototypical gifted person which is typically imagined to be a White or Asian male. In these studies, no effects for Whites, Asians or males were reported. So far, however, research in this area has only considered one identity – i.e., either gender *or* ethnic identity – in isolation.

In the present research, we investigate the intersection of gender and ethnicity. We hypothesize that the combination of gender and ethnicity – and specifically the extent to which the combination of these group memberships match the gifted White/Asian male prototype (Niedenthal et al., 1985; Bian et al., 2018) – may be relevant in determining the extent to which organizational fixed excellence theories make individuals belong: Fixed theories emphasizing giftedness rather than effort may signal prototypical White/Asian males are most likely to succeed. Accordingly, we hypothesized that fixed theories impair the belonging of individuals who do not match the gifted White/Asian male prototype, i.e., females from intellectually stereotyped ethnic minority communities, but even increase the sense of belonging of White/Asian males matching the prototype of the gifted.

### Individuals’ Sense of Belonging

As “social animals,” humans are driven by their need for belonging and social connection (Baumeister and Leary, 1995). Developing a strong sense of belonging in a given community can thus be seen as an important outcome in its own right. At the same time, individuals’ subjective sense of belonging has also been shown to be a crucial determinant of other important outcomes, as diverse as individuals’ engagement and performance in academic and professional settings, the formation of friendships and associated social capital, as well as mental and physical health (Walton and Cohen, 2007, 2011; Wilson et al., 2015; Yeager et al., 2016).

While the need for belonging seems to be largely universal (Baumeister and Leary, 1995; Walton et al., 2012), individuals seem to differ in the way they respond to different environmental factors regarding their sense of belonging. Overall, previous research suggests that maintaining a strong and stable sense of belonging seems to be more difficult for individuals who are negatively stereotyped in a given context. Negatively stereotyped individuals have been shown to experience higher fluctuations in their sense of (non)belonging in their daily lives and to doubt their belonging more readily when confronted with non-belonging cues, such as the experimentally induced perception that one might not have a lot of friends (Walton and Cohen, 2007; Yeager et al., 2016). Moreover, many public spaces seem to include cues that cater to the mainstream White male culture, but may impair the belonging of other individuals. Several studies (Fryberg, 2012; Fryberg et al., 2013; Brannon et al., 2015) have for example shown that universities primarily focus on independent values (e.g., stressing the importance of finding one’s own individual path for students), which can create the sense of a “cultural mismatch” and non-belonging for non-White and female individuals, who are culturally more attuned to interdependent values. Understanding how different student groups’ – and specifically negatively stereotyped individuals’ – sense of belonging can be strengthened is thus a crucial task, which we aim to pursue in this research through the means of organizational implicit theories.

### Organizational-Level Implicit Theories of Intelligence

Previous research on implicit theories has mostly focused on *individuals’* implicit theories or mindsets – i.e., the extent to which *a person* thinks that certain attributes like intelligence or skills are malleable (growth mindsets) or innate (fixed mindsets, Dweck, 2008). Building on this work, some recent research has begun to explore organization-level implicit theories – i.e., the extent to which *organizational* culture is broadly perceived to reflect a belief in the malleability or fixedness of certain attributes. Murphy and Dweck (2010) have highlighted the added value of organization-level implicit theories by showing how organizations – beyond their individual members’ mindsets – may themselves maintain distinct implicit theories. Specifically, Murphy and Dweck showed that organizational implicit theories, communicated through, for instance, advertising material or statements of organization members, lead members to adapt to these theories, reproducing them in the ways they see and present themselves, how they judge others, and how they select new employees. Through these top-down adaptation and selection processes, organizations may maintain distinct implicit theories on the long run.

A series of experiments by Emerson and Murphy (2015) has further shown organizational implicit theories to shape individuals’ attitudes toward organizations. Specifically, organizations with a malleable (versus fixed) theory of intelligence led individuals to think they would be judged more positively, feel more accepted, and exhibit more trust as well as engagement. While effects tended to be more pronounced for women, who may be more sensitive to the possibility of being judged negatively in business contexts (Kray and Shirako, 2011), they largely held for males, too. In line with research showing that growth mindsets of intelligence helps individuals see failures as opportunities for growth rather than a lack of talent (Dweck and Leggett, 1988; Smiley et al., 2016), organization-level malleable theories of intelligence seem to signal the respective institution to be more accepting and less judgmental toward employees and their mistakes.

Beyond the question to what extent intelligence is malleable or not, an equally crucial matter may be to what extent individuals’ excellent achievements – i.e., outstanding outcomes, rather than skills – are thought to be malleable: Excellent achievements can be thought of as being pre-determined and fixed by individuals’ innate intellectual giftedness as an extraordinarily high form of intelligence (fixed theory of excellence), or as being malleable, having to be developed through hard work and effort (malleable theory of excellence). Both theories of excellence seem wide-spread in organizations: Investigating service organizations’ online communication, Leung et al. (2020) found that around a quarter of investigated companies showed a pronounced fixed excellence theory, emphasizing giftedness, while another quarter showed a pronounced malleable focus, emphasizing effort; with the remaining organizations showing either a mixed theory (mentioning both talent and effort) or no indication of either theory.

Although innate giftedness is not commonly thought to be distributed differently in varying social groups (Steele and Aronson, 1995; Hyde, 2005; Penner, 2008), research suggests that the use of messages emphasizing the importance of giftedness may impair negatively stereotyped individuals’ sense of belonging. Specifically, Leslie et al. (2015) found evidence that faculty members’ domain-specific beliefs in the importance of giftedness were related to females’ and African Americans’ underrepresentation in the respective academic fields: The stronger faculty members endorsed that giftedness as an innate, fixed quality was the cornerstone of success, the fewer females and African Americans the respective domain seemed to attract.

Even more importantly, Bian et al. (2018) conducted several experimental studies in which success was portrayed as either requiring innate giftedness (corresponding to a fixed excellence view) or dedication and motivation (malleable view). They found that women’s interest and anticipated sense of belonging in various educational and professional opportunities was lower in fixed excellence organizations emphasizing giftedness rather than malleable organizations focusing on motivation, while no significant difference was found for males. Bian and colleagues also provided evidence for the idea that individuals’ match with prototypes may determine their sense of belonging: Specifically, they found effects on individuals’ sense of belonging in the respective organization to be explained by their perceived similarity to the prototypical organization member. In contrast, stereotype threat, i.e., women’s anticipation that they may be negatively stereotyped by others, did not mediate effects.

Relatedly, an experimental study by Rattan et al. (2018) showed that making individuals believe that only some people had what it takes to succeed negatively affected sense of belonging in females and negatively stereotyped ethnic minorities studying in science, technology, engineering, and math (STEM) fields. The view that only some individuals have the potential to succeed is implied in the fixed view of excellence, claiming that excellent achievements are innate and cannot be achieved by effort.

In sum, these findings suggest that compared to malleable theories of excellence, fixed theories have a negative effect on individuals whose gender or ethnicity does not match the gifted White/Asian male prototype, while no effects appear for individuals whose gender or ethnicity matches the gifted prototype.

This research has so far only investigated either gender *or* ethnicity, differentiating between individuals who match the gifted White/Asian male prototype with either their gender or ethnicity (i.e., males and White or Asian individuals) and individuals who do not match this prototype with their gender or ethnicity (i.e., females and negatively stereotyped ethnic minorities) only. However, every person carries both, a gender and an ethnic identity, at the same time. Accordingly, the gifted prototype is also characterized by both features, (male) gender and (White or Asian) ethnicity. Individuals’ match with the gifted prototype can therefore vary between a full prototype match regarding both group memberships (i.e., White males or Asian males), no matching group memberships (females from negatively stereotyped ethnic groups); and a mixed match (i.e., White females, Asian females, males from negatively stereotyped ethnic minority groups). Investigating the combined effects of gender and ethnicity, we consider all three degrees of self-prototype match in our study.

### Advantageous Effects of Fixed Implicit Theories in the Context of Positive Stereotypes

Despite the wide range of domains investigated, research on both individual mindsets and organizational-level implicit theories has so far focused on the *negative effects of fixed implicit theories or mindsets* on individuals (e.g., Chiu et al., 1997; Dweck, 2008; Murphy and Dweck, 2010; Emerson and Murphy, 2015; Yeager et al., 2019). Findings from two previous studies investigating how individuals’ intelligence mindsets impact their performance after relevant stereotypes have been activated indicate, however, that fixed views may not always carry universally negative effects, but even show advantageous effects for some individuals: Froehlich et al. (2016) as well as Mendoza-Denton et al. (2008) found that fixed intelligence mindsets increased detrimental stereotype threat effects on negatively stereotyped individuals’ performance, but increased advantageous stereotype lift effects on positively stereotyped individuals’ performance. It thus seems that fixed intelligence mindsets can function as moderators in context of stereotype-based effects, strengthening both, negative effects of negative stereotypes and positive effects of positive stereotypes.

Building on this line of work, we reasoned that previously found moderating effects of implicit theories may not be limited to individual-level implicit theories, but could extend to organizational implicit theories of excellence. We expected that students who do not match the gifted prototype with their gender and ethnicity (female ethnic minorities) experience impairments in their belonging by a fixed (vs. malleable) theory, while students with a full match (White/Asian males) benefit from a fixed versus malleable organizational-level implicit theory. Students with a mix of matching and non-matching group memberships (White/Asian females, males from negatively stereotyped ethnic minority groups) fall in between those two extremes and may thus show no overall effects. This latter assumption is supported by previous stereotype threat/lift research suggesting that, when no identity is experimentally activated, individuals with mixed prototype match may show no consistent stereotype-based effects: While individuals may suffer from the activation of a negatively stereotyped social identity (e.g., female gender), and profit from the activation of a positively stereotyped identity (e.g., Asian ethnicity; Shih et al., 1999), no consistent effects were found when both positive and negative identities were activated or when no identity was activated (Gonzales et al., 2002; Gresky et al., 2005; Rydell et al., 2009).

### The Present Research

As outlined, the present research aims to investigate whether organizational implicit theories of excellence may differentially affect individuals’ belonging depending on the extent to which they match the prototype of the gifted White/Asian male. We investigated this issue using the original advertising material obtained from Germany’s biggest and most selective scholarship organization. This material is sent out to several thousand top performing university students each year (usually the top 2% of students, as assessed by their grades). We used the material in its original form for the fixed condition and constructed an analogous manipulated version for the malleable condition. All data and material can be found online: https://osf.io/r359f/?view_only=c7d654c2f5bf4953ad17954d5aa72244.

## Materials and Methods

### Participants

Participants were recruited at a university in Germany as well as online through email lists for students and German-speaking student groups on the social media platform Facebook. The study was conducted online and was said to investigate the experiences of students at their university. Participants could win Amazon vouchers worth 200 Euros. In total, 663 students completed our online questionnaire. The mean age was *M* = 24.27, SD = 5.36.

### Procedure

After giving informed consent, students were randomly assigned to one of two conditions (fixed or malleable) and subsequently provided with the respective version of scholarship advertising material. After reading the respective material, they completed a questionnaire with our outcome variable (the belonging measure) and demographic information.

### Experimental Manipulation

The information material describes the services that the scholarship entailed (e.g., a 300 Euro monthly stipend, free seminars on diverse topics in- and outside of Germany, scholarships to study abroad) as well as information about requirements for successful applications.

The original version of the advertising material served as the fixed condition. With minor exceptions (we, e.g., changed the name of the scholarship organization to ensure that prior associations with the well-known organization would not affect results), no changes were made.

To create material for the malleable condition, we manipulated only the four expressions in the 225-word document which referred to implicit theories of excellence. Following previous implicit theory manipulations (e.g., Chiu et al., 1997; Mendoza-Denton et al., 2008; Yeager et al., 2019), the malleability condition emphasized that excellence must be developed through effort and diligence, while the fixed condition emphasized innate giftedness as the most important characteristic of their successful applicants. The crucial fixed (vs. malleable, in brackets) manipulation material reads as follows (manipulated parts are underlined):

“The Bahde Foundation offers one of the largest German giftedness scholarship programs (/scholarship programs). Requirement profile: Under the motto “Performance, Initiative, and Responsibility,” the Bahde Foundation supports young people with high scientific or artistic talent (/high commitment) who, guided by curiosity and a passion for knowledge, successfully study and conduct research (/continuously advance in their studies and research through diligence), develop and implement ideas on their own initiative, actively engage themselves beyond their own concerns – and from whom special achievements in the service of the general public can therefore be expected according to their talent (/extraordinary willingness to work hard) and personality.”

### Measures

#### Prototype Match Regarding Group Membership

We assessed individuals’ ethnicity and gender to determine the extent to which their group memberships matched the prototype of the gifted White/Asian male. Individuals were asked to indicate their gender, and whether their parents or grandparents came from a country other than Germany (a question commonly used in Germany as a replacement for more direct questions about ethnicity or race; e.g., German Federal Statistical Office, 2005). The degree of match with the gifted prototype was coded as the number of matching group memberships regarding gender (male = fit, female = non-fit) and ethnicity (White or Asian = fit, non-Asian minority = non-fit), with White or Asian males showing the highest match (two matching group memberships) and females from negatively stereotyped ethnic minority groups showing the lowest match (0 matching group memberships).

Overall, 70% of participants (467) were female. Twenty-three% (153) were members of non-Asian ethnic minority groups. Of the remaining 77% (510) of participants, nine indicated being from an Asian background and 501 to be White. Table 1 includes information on the number of participants by prototype match and condition. In sum, 21% of participants matched the gifted prototype with their gender and ethnicity (i.e., indicating male gender as well as White or Asian ethnicity), 64% held one matching identity (i.e., indicating either male gender or White/Asian ethnicity) and 15% held no matching group identities (i.e., indicating female gender and non-Asian ethnic minority status).

**TABLE 1:** Descriptive statistics of individuals’ anticipated sense of belonging by condition and prototype match.

Condition	Prototype Match	*M*	*SD*	*N*
Fixed theory	No match	3.30	1.20	50
	Mixed match	3.34	1.33	216
	Match	3.78	1.23	73
Malleable theory	No match	4.04	1.45	49
	Mixed match	3.28	1.37	205
	Match	3.28	1.30	69

*Prototype match = degree to which individuals’ social identities (gender and ethnicity) match the gifted White/Asian male prototype (match = White/Asian males; no match = females from stereotyped ethnic minority groups; mixed match = White/Asian females and males from stereotyped ethnic minority groups).*

#### Manipulation Check

To check whether our manipulation successfully manipulated individuals’ perception of the organization’s implicit theories, participants were asked in how far the attributes “gifted” and “intelligent” (α = 0.71) applied to a typical scholarship holder (1 = “does not apply at all,” 7 = “fully applies”).

#### Anticipated Belonging

To assess participants’ anticipated belonging with the foundation, students were asked how much they agreed to the following two items modeled after existing scales (Walton and Cohen, 2007; Murphy and Zirkel, 2015): “I think I would feel like I belong at the Bahde Foundation” and “I think I am the kind of person the Bahde Foundation is looking for” (1 = “strongly disagree”, 7 = “strongly agree”; α = 0.80).

#### Prior Achievement

To control for individuals’ prior achievement, we asked students to indicate the grades they received on their three last exams. These grades were than averaged to one prior-achievement score. In order to prevent the 22 students (3%) who did not complete this measure (possibly because they did not receive any grades yet) from being excluded, we imputed mean scores for these students. The mean GPA was *M* = 1.93 (corresponds to the letter B in the United States-American system).

## Results

### Manipulation Check

We used an ANOVA to check, if our manipulation changed participants’ perception of the organizations’ implicit theories of excellence. Results revealed a significant condition effect, *F*(1,661) = 20.47, *p* < 0.001, η*^2^* = 0.030. Participants imagined a typical scholarship holder to be more gifted in the fixed condition (*M* = 5.64, *SD* = 0.96) than in the malleable condition (*M* = 5.28, *SD* = 1.07).

### Anticipated Belonging

To check whether the effect of implicit theories would vary with individuals’ degree of match with the prototype of the gifted White male student, we conducted several ANOVAs controlling for individuals’ prior achievement. Means and standard deviations for participants’ sense of belonging by prototype match and condition are reported in Table 1.

We first conducted a 2 (condition) × 3 (degree of prototype match) ANOVA. As expected, we found a prototype match × condition interaction on anticipated belonging, *F*(2,655) = 6.10, *p* = 0.002, η*^2^* = 0.018, suggesting that the effect of organizational implicit theories indeed varies with the extent to which individuals match the gifted prototype.

**FIGURE 1:**
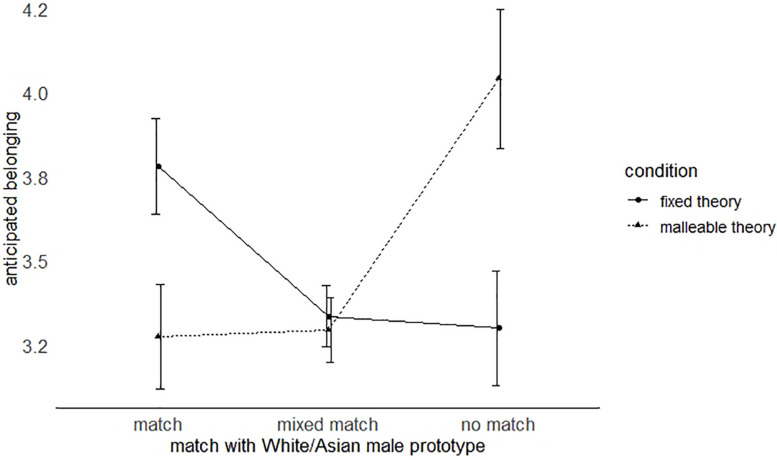
Effects of fixed versus malleable theories of excellence on individuals’ anticipated sense of belonging depending on the extent their social identities match the gifted prototype. Match = White or Asian males; no match = females from stereotyped ethnic minority groups; mixed match = White or Asian females and males from stereotyped ethnic minority groups. Error bars represent ±1 standard error.

*Post hoc* ANOVAs testing the condition effect for the different subgroups further confirmed hypotheses, as illustrated in Figure 1: Individuals matching the gifted prototype – i.e., White/Asian males – anticipated higher belonging in the organization with a fixed view of excellence than in the organization with a malleable view, *F*(1,139) = 4.64, *p* = 0.032, η*^2^* = 0.033. Conversely, individuals whose personal group memberships did not match the gifted prototype – i.e., females from negatively stereotyped ethnic minority groups – anticipated higher belonging in the malleable organization than the fixed condition, *F*(1,96) = 7.66, *p* = 0.007, η*^2^* = 0.074. Groups of individuals with a mixed prototype match in group memberships – i.e., White/Asian females and males from stereotyped ethnic minority groups – showed no significant difference between fixed and malleable condition overall, *F*(1,419) = 0.12, *p* = 0.732, η*^2^* < 0.001 [*F*(1,365) = 0.007, *p* = 0.934, η*^2^* < 0.001 and *F*(1,51) = 0.95, *p* = 0.334, η*^2^* = 0.018, respectively].

Regarding main effects, we found a non-significant main effect of the experimental manipulation, *F*(1,655) = 0.33, *p* = 0.57, and a significant main effect of prototype match, *F*(2,655) = 5.44, *p* = 0.005, η*^2^* = 0.016. Exploratory *post hoc* analyses suggest that overall, individuals with a mixed prototype match exhibited lower levels of belonging than individuals with a full match, *p* = 0.020, and individuals with no match, *p* = 0.005. Individuals with a full and no match did not differ significantly, *p* = 0.520. While individuals with no match experienced an increase in belonging in the malleable condition, and individuals with a full match experienced an increase in belonging in the fixed condition, individuals with a mixed match showed similarly low levels of belonging in both conditions.

## Discussion

Selective academic organizations like the Ivy League or scholarship providers strive for individuals with excellent academic achievements – presumably independent of individuals’ demographics. Yet, frequently used fixed messages of excellence, emphasizing innate giftedness as criterion for individuals’ excelling in selective academic institutions may signal that only a certain group of people – specifically individuals who match the prototype of the gifted White/Asian male – can belong at the respective organization.

Previous research in this area has focused on either females or negatively stereotyped ethnic minorities, suggesting that fixed excellence messages may impair their belonging, while others (i.e., White/Asian students and males) may be unaffected. Investigating the combined effects of gender and ethnicity in a relatively large student sample, we expected the extent to which organizations’ implicit theories of excellence make individuals feel they belong to depend on the degree to which individuals’ gender and ethnicity match the gifted White/Asian male prototype. Consistent with our hypotheses we found that individuals who do not match that prototype – i.e., females from negatively stereotyped ethnic minority groups – showed a reduced sense of belonging in the organization conveying a fixed (vs. malleable) implicit theory of excellence. In contrast, White and Asian males, who fit the prototype of the gifted, benefited regarding their sense of belonging in the organization with a fixed view of excellence. Students with a mix of matching and non-matching group memberships (i.e., White or Asian females and males from stereotyped ethnic minorities) exhibited similar levels of belonging in both conditions in line with the idea that the opposing effects of the matching and mismatching identity may cancel each other out (Gonzales et al., 2002; Gresky et al., 2005; Rydell et al., 2009).

### Contributions to Theory and Practice

This research makes important contributions to theory and practice. First, our results highlight how organizational fixed implicit theories may not always carry negative effects for all people, but may even serve certain groups in some specific contexts. Previous research has almost exclusively focused on the negative effects of fixed mindsets or implicit theories. Complementing two earlier studies about the advantageous effects of fixed intelligence *mindsets* on positively stereotyped individuals in stereotype lift paradigms (Mendoza-Denton et al., 2008; Froehlich et al., 2016), we show that fixed *organizational theories*, too, and can yield advantageous effects for individuals who match the gifted prototype. Overall, our results support the assumption that implicit theories can moderate effects of stereotypes, with fixed theories strengthening both negative effects of negative and positive effects of positive stereotypes. Importantly, this does not mean that fixed theories are always beneficial for positively stereotyped individuals. There is for example little reason to assume that the negative effects of fixed intelligence mindsets on individuals’ response to failure (Dweck and Leggett, 1988; Smiley et al., 2016) would not also apply to positively stereotyped individuals. Only in specific stereotype-relevant contexts, fixed theories may be beneficial to positively stereotyped individuals. We hope our results help develop a more nuanced understanding of the effects fixed and malleable theories may carry for diverse individuals and contexts.

Second, in examining the intersection of multiple social identities, our research highlights the usefulness of this approach when investigating organizational implicit theories. Many scholars in social psychology have called for research to study the interplay of different social identities (e.g., McCall, 2005; Purdie-Vaughns and Eibach, 2008; Cole, 2009), and emerging empirical results highlight the importance of such intersectional approaches, illustrating that the combined effects of two identities can play out in different ways which cannot be predicted from separate investigations into each identity – in some cases adding up, and in other cases showing interactive effects (Shih et al., 1999; Levin et al., 2006; Purdie-Vaughns and Eibach, 2008). Still, relatively little research has so far done so. With respect to organizational implicit theories, there is to our knowledge no research taking an intersectional approach. Compared to previous research on the effects of organizational excellence theories or similar constructs, which only investigated one social identity (gender or ethnicity) in isolation and has only found negative effects for negatively stereotyped individuals, the intersectional approach in the present research yielded a more nuanced picture of results for different subgroups depending on the degree to which their identities match the gifted prototype.

Third, our research highlights the practical importance of organizational implicit theories of excellence in shaping students’ anticipated belonging to academic institutions. The present research used the original and a manipulated version of advertising material obtained from Germany’s biggest and most selective scholarship organization and investigated a sample of relatively high performing students as a potential target group for the scholarship organization. In doing so, we highlight how currently used fixed excellence messages may impair the belonging of negatively stereotyped individuals in academic institutions and how conversely, the (tailored) use of a malleable view on excellence could help make underrepresented female ethnic minority students feel like they belong.

### Questions for Future Research

Our research also raises exciting questions for future research to investigate. Firstly, future research should compare different operationalizations of malleable excellence theories as well as malleable intelligence theories. While our malleable excellence condition emphasized the importance of effort and hard work, other excellence theory research has previously used concepts more closely related to motivation and dedication in their malleability treatments (Bian et al., 2018). Emphasizing the importance of effort may imply that successful candidates have to be strongly motivated, too, but compared to a motivation focus it may go a step further in stressing that the implementation of motivation into goal-oriented behavior is also required. It is thus conceivable that the motivation-focus in malleable theories may be perceived as less demanding than the effort focus and thus elicit more positive responses. Similarly, malleable theories of *intelligence* conveying that every organization member can become smarter may signal a higher tolerance for mistakes (Smiley et al., 2016) and appear less demanding than malleable excellence theories stressing that a high degree of effort is required. This may explain why some studies have found positive effects of malleable excellence and intelligence theories on individuals’ belonging more broadly, and not only for women from ethnic minority groups, as in our study (Emerson and Murphy, 2015; Bian et al., 2018).

Secondly, with regards to the mixed prototype match group (e.g., White males), future research should explore if the activation of individuals’ positively versus negatively stereotyped identity moderates the effects of organizational excellence theories on their sense of belonging. In our study, we did not specifically activate any identity. With this approach, we did not find any condition effect for individuals with a mixed prototype match. Previous research on stereotype threat and stereotype lift effects suggests that a targeted activation of individuals’ positive vs. negative identity can elicit positive stereotype lift vs. detrimental stereotype threat effects, respectively (Shih et al., 1999; Rydell et al., 2009). Accordingly, such a targeted identity activation (or even a chronic activation of a certain social identity which may be present in some populations) may also shape the effect of organizational implicit theories on belonging.

Thirdly, as a basis for deriving interventions to support organizations in developing a malleable culture of excellence, it would be interesting to explore how exactly implicit theories of excellence emerge. One possible explanation of how fixed theories arise would be that the people in power who create them are often White/Asian males, to whom the fixed messages may be more appealing than to other groups. Another possibility is that fixed messages may broadly, irrespective of individuals’ gender or ethnicity, seem more exclusive and thus desirable for individuals who have already joined an organization. Being part of a group of naturally gifted individuals, born with innate talents, may seem to be more special and appealing than being part of a diligent, hardworking group. Understanding how implicit theories of excellence emerge in the first place may help develop targeted interventions that could optimize organization’s implicit theories in the long run.

Furthermore, future research should explore consequences of organizational excellence theories on individuals beyond belonging. Previous research has associated individuals’ sense of belonging in a given environment with diverse outcomes such as individuals’ engagement, performance, and social integration (Walton and Cohen, 2007, 2011; Wilson et al., 2015; Yeager et al., 2016). Would fixed messages of excellence, in line with this research, undermine these outcomes for stereotyped individuals, leading them to, e.g., disengage in completing their application material, show worse performance in assessment center tests or being less sociable around members of the respective organization? Would adverse effects on negatively stereotyped individuals’ belonging and related outcomes also show after individuals may have obtained a scholarship and thus impair their experience and engagement within the organization? And would the same effects show for White/Asian males receiving malleable messages of excellence or would their outcomes be buffered against such impairments by the previously reported heightened long-term stability in their sense of belonging (Walton and Cohen, 2007)?

Finally, it would be interesting to explore boundary conditions of our effects. Our findings emerged in cultures in which giftedness is strongly associated with White/Asian males rather than females or non-Asian ethnic minorities. While we are not aware of any (sub)cultures in which these associations are not common, it may be interesting to experimentally change individuals’ associations between giftedness and different demographic groups and investigate, if a more inclusive sense of giftedness (i.e., giftedness being less strongly associated with White/Asian males) could reduce differential effects and make individuals feel they belong more equally irrespective of fixed vs malleable excellence messages.

## Data Availability Statement

The datasets presented in this study can be found in an online repository under the following link: https://osf.io/r359f/?view_only=c7d654c2f5bf4953ad17954d5aa72244.

## Ethics Statement

The studies involving human participants were reviewed and approved by Ethikkommission der Freien Universität Berlin. The patients/participants provided their written informed consent to participate in this study.

## Author Contributions

BH and CB designed and conducted the research and wrote the manuscript. CB conducted the analyses. Both authors contributed to the article and approved the submitted version.

## Conflict of Interest

The authors declare that the research was conducted in the absence of any commercial or financial relationships that could be construed as a potential conflict of interest.
